# Supplementary material for: Augmented hip proprioception influences mediolateral foot placement during walking
Source: IEEE Trans Neural Syst Rehabil Eng. Author manuscript; Available in PMC 2026 Jun 23. (PMC13289793; doi:10.1109/TNSRE.2021.3114991)
Supplement: Supplementary Material [file NIHMS2182745-supplement-Supplementary_Material.pdf]

Supplementary Material for "Augmented hip proprioception influences mediolateral foot placement during walking"

Holly A. Knapp, Blaire A. Sobolewski, Jesse C. Dean

APPENDIX A

THIS study used R^2 values calculated from linear regressions to quantify the strength of the relationship between pelvis dynamics and mediolateral stepping behavior. We calculated the proportion of the variation in mediolateral foot placement, final pelvis displacement, and step width that was predicted by the combination of mediolateral pelvis displacement and velocity. Here, we instead use partial correlations to focus on the separate contributions of pelvis displacement and pelvis velocity to the observed stepping behavior. The 10 quantified partial correlation metrics are listed in Table SI with corresponding brief descriptions.

As in the main text, statistical comparisons were performed separately for Experiment 1 and Experiment 2. We used repeated measures one-way ANOVA to determine whether each partial correlation metric differed across the four feedback conditions. In the case of a significant effect, we used Tukey-Kramer post-hoc tests to identify significant differences between individual conditions. For all comparisons, p-values less than 0.05 were interpreted as significant.

The partial correlation between mediolateral pelvis displacement and several metrics of stepping behavior differed across feedback conditions, paralleling our main text results investigating R^2 magnitude. Step start ρ_{disp_FP} varied significantly across conditions (Fig. S1a) for both Experiment 1 ($p=0.0002$) and Experiment 2 ($p<0.0001$), and was highest for the Augmented feedback condition. Step end ρ_{disp_FP} also varied significantly across conditions (Fig. S1b) for both Experiment 1 ($p=0.02$) and Experiment 2 ($p=0.0005$), and was highest with Augmented feedback. Step start ρ_{disp_PD} did not vary across conditions (Fig. S1c) for either Experiment 1 ($p=0.97$) or Experiment 2 ($p=0.17$). Step start ρ_{disp_SW} varied significantly across conditions (Fig. S1d) for both Experiment 1 ($p=0.049$) and Experiment 2 ($p=0.001$), and was highest for the Augmented feedback condition. Step end ρ_{disp_SW} did not vary across conditions (Fig. S1e) for either Experiment 1 ($p=0.29$) or Experiment 2 ($p=0.25$).

In contrast to our observations with pelvis displacement,

TABLE SI
PARTIAL CORRELATION METRICS

Metric	Description
step start ρ_{disp_FP}	Partial correlation between mediolateral pelvis displacement at the start of a step and mediolateral foot placement at the end of the step, accounting for mediolateral pelvis velocity at the start of the step
step end ρ_{disp_FP}	Partial correlation between mediolateral pelvis displacement at the end of a step and mediolateral foot placement at the end of the step, accounting for mediolateral pelvis velocity at the end of the step
step start ρ_{disp_PD}	Partial correlation between mediolateral pelvis displacement at the start of a step and mediolateral pelvis displacement at the end of the step, accounting for mediolateral pelvis velocity at the start of the step
step start ρ_{disp_SW}	Partial correlation between mediolateral pelvis displacement at the start of a step and step width at the end of the step, accounting for mediolateral pelvis velocity at the start of the step
step end ρ_{disp_SW}	Partial correlation between mediolateral pelvis displacement at the end of a step and step width at the end of the step, accounting for mediolateral pelvis velocity at the end of the step
step start ρ_{vel_FP}	Partial correlation between mediolateral pelvis velocity at the start of a step and mediolateral foot placement at the end of the step, accounting for mediolateral pelvis displacement at the start of the step
step end ρ_{vel_FP}	Partial correlation between mediolateral pelvis velocity at the end of a step and mediolateral foot placement at the end of the step, accounting for mediolateral pelvis displacement at the end of the step
step start ρ_{vel_PD}	Partial correlation between mediolateral pelvis velocity at the start of a step and mediolateral pelvis displacement at the end of the step, accounting for mediolateral pelvis displacement at the start of the step
step start ρ_{vel_SW}	Partial correlation between mediolateral pelvis velocity at the start of a step and step width at the end of the step, accounting for mediolateral pelvis displacement at the start of the step
step end ρ_{vel_SW}	Partial correlation between mediolateral pelvis velocity at the end of a step and step width at the end of the step, accounting for mediolateral pelvis displacement at the end of the step

Definitions of various partial correlation values used to quantify the contributions of pelvis displacement and velocity to stepping behavior

none of the partial correlations focused on pelvis velocity varied significantly across feedback conditions ($p \geq 0.14$ for all comparisons). Values are illustrated in Figure S2 for visual comparison.

The present results indicate that the changes in R^2 magnitude described in the main text are predominantly due to a change in the step-by-step relationship between pelvis displacement and stepping behavior, and not to a change in the relationship between pelvis velocity and stepping behavior. The relative importance of pelvis displacement is likely caused in part by our choice to control vibration intensity based on pelvis displacement, not pelvis velocity.

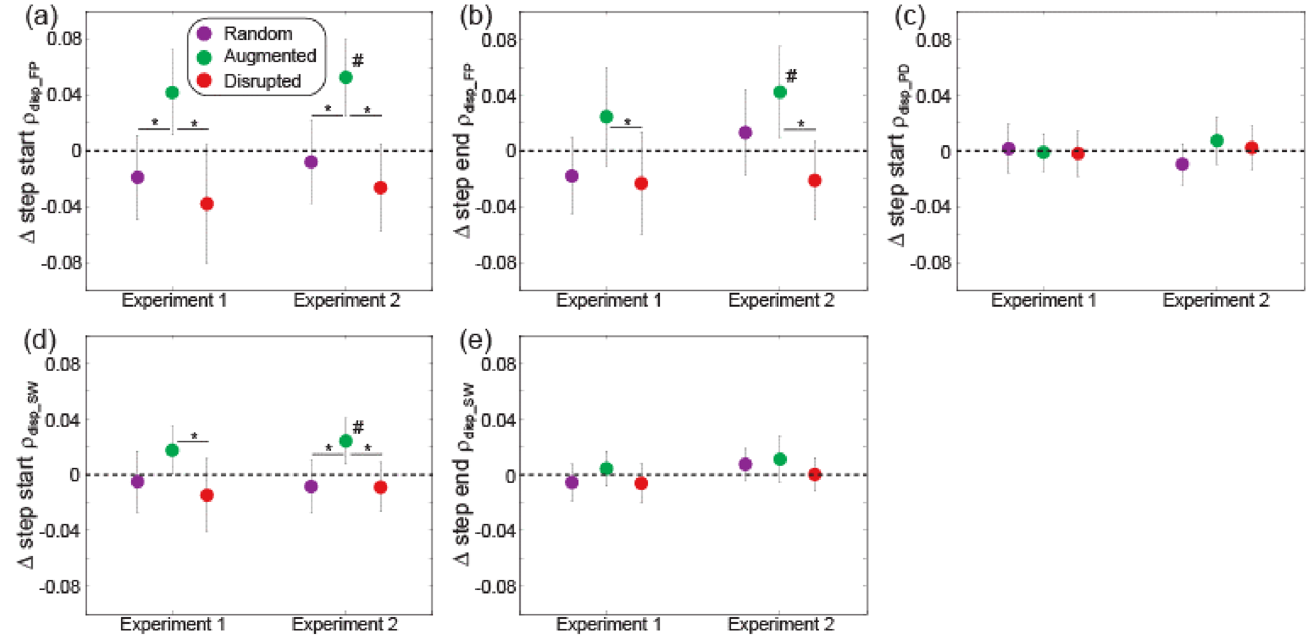

Fig. S1. Several metrics based on the partial correlation with mediolateral pelvis displacement varied across feedback conditions. As in the main text, we illustrate the change in the partial correlation metrics relative to the No feedback condition. We illustrate the change in step start ρ_{disp_FP} (a), step end ρ_{disp_FP} (b), step start ρ_{disp_PD} (c), step start ρ_{disp_SW} (d), and step end ρ_{disp_SW} (e). Data points indicate means and error bars indicate 95% C.I. Asterisks (*) indicate significant post-hoc differences between the indicated conditions, and pound signs (#) indicate significant post-hoc differences from the No feedback condition.

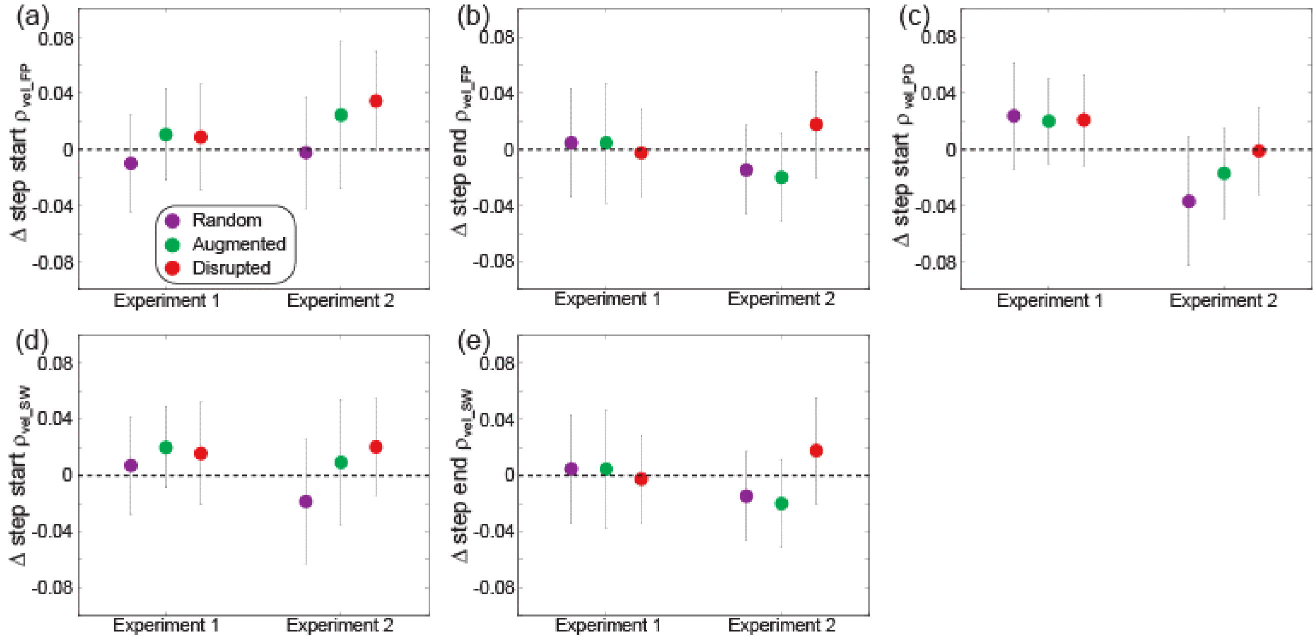

Fig. S2. Metrics based on the partial correlation with mediolateral pelvis velocity did not vary across feedback conditions, as illustrated relative to the No feedback condition. We illustrate the change in step start ρ_{vel_FP} (a), step end ρ_{vel_FP} (b), step start ρ_{vel_PD} (c), step start ρ_{vel_SW} (d), and step end ρ_{vel_SW} (e). Data points indicate means and error bars indicate 95% C.I.

APPENDIX B

This study primarily investigated the effects of hip vibration on the relationship between pelvis motion and foot placement, which is generally accepted as an important contributor to walking balance. For Experiment 2, we included a secondary exploratory analysis of the effects of hip vibration on metabolic rate. Our interest in this topic is based on the relationship between metabolic rate and step width. Briefly, metabolic rate increases when walking with steps that are either substantially wider or narrower than the preferred width [1]. Additionally, metabolic rate increases when the normal foot placement strategy is disrupted – for example, by visually prescribing foot placement location on a step-by-step basis [2]. Therefore, we sought to test whether the potential effects of hip vibration on the control of mediolateral foot placement were accompanied by changes in metabolic rate.

As described in more detail in the main text, we expected that Augmented feedback would strengthen the relationship between pelvis motion and foot placement, whereas Disrupted feedback would weaken this relationship. Following on that expectation, we hypothesized here that by promoting a stronger link between mediolateral foot placement and pelvis motion, Augmented feedback would decrease the use of unnecessarily costly wide or narrow steps, thus reducing metabolic rate. Conversely, we hypothesized that Disrupted feedback would increase the incidence of costly mechanically-inappropriate steps, and increase metabolic rate.

We used a portable metabolic cart (k4b2; Cosmed; Rome, Italy) to measure oxygen consumption and carbon dioxide production during each 6-minute walking trial in Experiment 2 (No feedback; Random feedback; Augmented feedback; Disrupted feedback). Participants rested for at least 3-minutes between trials. A standard equation was used to calculate metabolic rate for each 30-second period [3], and metabolic rate was normalized by participant body mass. To focus on the net metabolic cost due to walking, we subtracted the average metabolic rate during the final 3-minutes of an initial 6-minute trial in which participants stood quietly.

Our initial statistical analysis investigated whether the different feedback conditions influenced metabolic rate, as well as whether any such effects varied with time. We performed a two-way repeated measures ANOVA with interactions, with the independent variables of feedback condition (No feedback; Random feedback; Augmented feedback; Disrupted feedback) and time (each 30-second bin). The dependent variable was metabolic rate. Tukey-Kramer post-hoc tests were performed as appropriate.

Metabolic rate was clearly influenced by time ($p<0.0001$), following the typical pattern of increasing for approximately the first two minutes of walking before reaching a plateau (Fig. S3a). Metabolic rate was also significantly affected by feedback condition ($p=0.017$). For purposes of visual clarity, the average metabolic rate across the entire 6-minute walking trial is illustrated for each feedback condition in Figure S3b. Metabolic rate was highest for the Augmented condition, although the only post-hoc comparison that reached statistical significance was a 3% difference between the Augmented and Random feedback conditions (Fig. S3b).

Despite the aforementioned statistical significance, visual inspection of Figure S3a suggested that the elevated metabolic rate with Augmented feedback was most evident early in the walking trial. Therefore, in a follow-up analysis, we restricted the comparison time period to the last 3-minutes of the walking trials (a typical "steady-state" period) across the four feedback conditions. This comparison of steady-state metabolic rate did not reveal a significant effect of time ($p=0.22$), feedback condition ($p=0.07$), or an interaction between time and feedback condition ($p=0.79$) (Fig. S3c).

Whether focused on the entire 6-minute walking trial or just the steady-state period, our analyses of metabolic rate did not support our hypotheses. We found no evidence that Augmented feedback decreased metabolic rate – which we hypothesized would occur by allowing participants to walk with steps that were "just right" for the dynamic state of the pelvis, and avoid the increased costs of steps that were overly wide or narrow. Similarly, we found no evidence that Disrupted feedback increased metabolic rate, as we hypothesized would be caused by an increased prevalence of inappropriate step widths.

The present results are equivocal with respect to whether Augmented feedback actually caused an *increase* in metabolic rate. No such significant effect was present when focused only on the steady-state period, but was observed when the entire

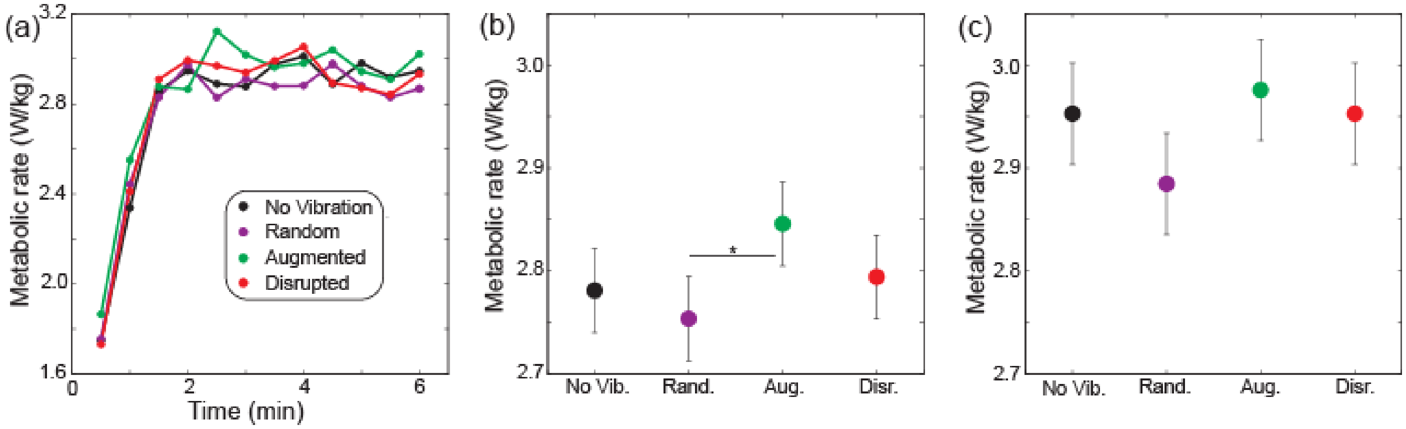

Fig. S3. Metabolic rate rapidly increased at the start of walking trials before reaching a plateau, as illustrated for the average values during each 30-second bin for each walking condition (a). Error bars are not included in panel a because of extensive overlap. Metabolic rate across the entire 6-minute trials exhibited a significant difference between conditions (b) while metabolic rate restricted to the steady-state period (final 3-minutes) did not (c).

walking trial was included in the analysis (albeit only when Augmented feedback was compared to Random feedback). While it is typical to focus only on the steady-state period, the dynamic response to exercise onset has allowed unique insight into differences in metabolic rate, such as changes that occur with increased age [4]. Future work using more sophisticated methods of quantifying metabolic rate during short time periods (such as in [5]) would be needed to reveal whether Augmented feedback truly causes an increased metabolic rate in the early walking phase. Speculatively, such an effect could be due to participants initially perceiving the Augmented feedback as reflecting an increased risk of a loss of balance, as may be caused by stance leg vibration eliciting a perception of the pelvis moving lateral to the stance leg. Participants may respond with a strategy of co-contracting the hip musculature to stabilize the pelvis on the stance leg and prevent this from occurring. Such a strategy could increase metabolic rate without being evident from kinematic measurements.

References

[1] J. M. Donelan, R. Kram, and A. D. Kuo, “Mechanical and metabolic determinants of the preferred step width in human walking.,” *Proc. Biol. Sci.*, vol. 268, no. 1480, pp. 1985–1992, Oct. 2001, doi: 10.1098/rspb.2001.1761.

[2] D. Wezenberg, A. de Haan, C. A. M. van Bennekom, and H. Houdijk, “Mind your step: Metabolic energy cost while walking an enforced gait pattern,” *Gait Posture*, vol. 33, no. 4, pp. 544–549, Apr. 2011, doi: 10.1016/j.gaitpost.2011.01.007.

[3] J. M. Brockway, “Derivation of formulae used to calculate energy expenditure in man,” *Hum. Nutr. Clin. Nutr.*, vol. 41, no. 6, pp. 463–471, Nov. 1987.

[4] M. A. Babcock, D. H. Paterson, D. A. Cunningham, and J. R. Dickinson, “Exercise on-transient gas exchange kinetics are slowed as a function of age,” *Med. Sci. Sports Exerc.*, vol. 26, no. 4, pp. 440–446, Apr. 1994.

[5] J. C. Selinger and J. M. Donelan, “Estimating instantaneous energetic cost during non-steady-state gait,” *J. Appl. Physiol. Bethesda Md 1985*, vol. 117, no. 11, pp. 1406–1415, Dec. 2014, doi: 10.1152/japplphysiol.00445.2014.
